# Representations of Free-Living and Unrestrained Dogs as an Emerging Public Health Issue in Australian Newspapers

**DOI:** 10.3390/ijerph18115807

**Published:** 2021-05-28

**Authors:** Chris Degeling, Julie Hall, Lily M. van Eeden, Summer M. Finlay, Suk Maya Gurung, Victoria J. Brookes

**Affiliations:** 1 Australian Centre for Health Engagement, Evidence and Values, University of Wollongong, Wollongong, NSW 2522, Australia; juliehal@uow.edu.au; 2 Department of Environment, Land, Water and Planning, Arthur Rylah Institute, Heidelberg, VIC 3084, Australia; lily.vaneeden@sydney.edu.au; 3 School of Life and Environmental Sciences, Faculty of Science, The University of Sydney, Camperdown, NSW 2006, Australia; 4 School of Health and Society, University of Wollongong, Wollongong, NSW 2522, Australia; sfinlay@uow.edu.au (S.M.F.); smmg069@uowmail.edu.au (S.M.G.); 5 School of Animal and Veterinary Sciences, Faculty of Science, Charles Sturt University, Wagga Wagga, NSW 2650, Australia; vbrookes@csu.edu.au; 6 Graham Centre for Agricultural Innovation (NSW Department of Primary Industries and Charles Sturt University), Wagga Wagga, NSW 2650, Australia

**Keywords:** Australia, media analysis, animal control, dog bites, social policy, public health

## Abstract

That dogs can live and breed as free-living animals contributes to public health risks including zoonotic transmission, dog bites, and compromising people’s sense of safety in public spaces. In Australia, free-living dog populations are comprised of domestic dogs, dingoes, and dog–dingo hybrids, and are described using various terms (for example, stray or community), depending on social or geographic context. Urban expansion and regional migration mean that risks associated with contact between humans and free-living dogs are increasing. Public health authorities, local governments, and community organisations have called for transdisciplinary partnerships to address dog-related health risks with a sustainable long-term approach. Values pluralism and a lack of sustained community engagement in affected areas have meant that the outcome of such efforts to date has been mixed. To identify ideas in public circulation about the impact of unrestrained and free-living dogs on human health and well-being, and understand the framework through which these animals are problematised and solutions are proposed in public discourse, we systematically examined coverage of these issues in print media. Our analyses indicate that reporting in Australian newspapers tends to frame the public health impacts of free-living dogs as problems of public order requiring direct government action to re-establish control. The public health impacts of free-living dog populations in Australia have complex causes that intersect at the nexus between human and canine behaviour, agricultural and land management practices, local bylaws, and efforts to conserve ecological systems. Placing responsibility on governments limits opportunities for greater community involvement in developing integrated One Health approaches. Better-quality evidence of the impacts of dog populations on community health and well-being, and broad community support are needed to reshape public debates on animal control, which, ultimately, will promote more effective approaches to mitigate dog-related public health risks at the human–animal–environment interface.

## 1. Introduction

Domestic dogs and humans are companion species—everywhere people reside in large numbers, dogs do too [1]. More than 4 million domestic dogs live as pets in Australia [2], with a significant population of native wild dogs (dingoes) also living freely in peri-urban, rural and remote areas [3,4]. The wider impacts of dog populations on human health in Australia have been described as an unrecognised and potentially growing public health problem [5,6]. Data about dog bites treated in primary care are not systematically reported but Australian national records suggests that the incidence of hospitalizations due to dog bites is increasing to more than 2000 persons per year, with increased incidence in rural/remote and socially and economically disadvantaged settings [6]. The ability of dogs to live and breed as free-living animals in and around human settlement contributes to a number of further public health risks including the potential transmission of zoonoses and compromising the safety of people in public spaces [5,7]. As well as being unsettling, uncontrolled dog behaviour and aggression can also have negative impacts on community cohesion, and how people use and share local streets and parks, by stealing food, spreading garbage, and increasing noise and faecal pollution [8,9,10]. Free-living dogs can have broader impacts through predation of livestock and wildlife—the loss of which can cause financial and ecological losses and emotional distress [11,12]. Increased numbers of free-living dogs can also affect the welfare of the dogs themselves. There is more competition for food, increased frequency of fights, potential for large outbreaks of disease and dangerous pack behaviour [13,14].

Since the mid-1990s, there has been a concerted effort by Australian governments to implement a strategic approach to managing both owned [15] and wild dog populations [16]. Because the outcomes of such efforts have been mixed, and in the case of wild dogs, any positive impacts are often short lived, public health authorities, local governments, not-for-profit organisations and community members have called for transdisciplinary partnerships to understand and address dog-related health risks with a sustainable, long-term approach [17,18]. In urban environments, animal bylaws, veterinary care and the norms of responsible dog keeping work to promote animal welfare and minimize the risks to human health and well-being from canine cohabitation [19,20]. Yet dog bites, dog aggression and other health impacts and risks associated with free-living dogs remain neglected in Australian health care and public health. One Health approaches to the human–animal–environment interface point to the potential to enhance health promotion and protection by focusing on the interactions between dog populations, people, and efforts towards community consultation and animal control [19,21]. At the core of One Health is the recognition that the failure to consider how humans relate to non-human animals and their shared environment can limit opportunities to enhance human and non-human health and well-being. However, interventions that aim to promote healthy coexistence between free-living dogs and humans can only succeed if they incorporate and respond appropriately to social values through sustained and coordinated community action [22,23]. 

Scientific and broader socio-cultural disagreement about the characteristics of different types of canids living in Australia also complicates management efforts [24]. Table 1 shows the characteristics of common descriptions of different categories of dogs found in Australia. With regard to owned dogs, in Australia, the norms of responsible ownership depend on context such that most dogs in urban environments are confined on private property and are accompanied by an owner and restrained (by a leash, for example) whenever they leave that property. However, in some rural and remote settings, it is common practice for owned dogs to be allowed to roam freely, unsupervised by their owners, including community or camp dogs that live in First Nations’ communities [10]. An unrestrained dog in any environment (urban, rural or remote) may also be a stray if they are recently lost or abandoned. “Wild dogs” is an umbrella term that is often used in Australia to describe different types of unowned dogs including dingoes, feral domestic dogs and dog–dingo hybrids [25]. Dingoes have been present in Australia for over 3500 years and are regarded as a native species under Australian environmental legislation [26,27], but their ability to hybridise with introduced domestic dogs, and the difficulty of accurately identifying hybrids, creates a grey area for defining wild dogs in Australia. Wild dog packs commonly comprise family groups residing in defined home ranges that vary greatly in size (from 45 to 115 km^2^) depending on the availability of food and water [28]. These resources are in relative abundance in and around human settlement such that hybridisation between domestic dogs and dingoes is far more common in rural and peri-urban areas [29]. Media analyses of the public health impacts of human–dog interactions in Canada and the United States point to geographic location as being a key determinate of how culpability for dog-related harms and responsibility for interventions are represented in this discourse [30,31]. In Australia, the cultural politics of and potential for an introduced species to hybridise with native fauna complicates ‘place-based’ analyses [32], even as the drivers of hybridisation and the incidence of problematic human-wild dog interactions overlap.

In both media depictions and policy discourses around health risks, how a problem is framed underpins public awareness and understanding, while also guiding and influencing the quality of the strategies and actions taken to address it [33,34,35]. Because elected officials, politicians and policy advisors are responsive to public opinion, public perceptions about the causes of a nuisance or threat might influence the level of public support for specific policies and proposed solutions, and, thereby, ongoing political debate [36,37]. In this article, our goals are to identify ideas in public circulation about the impact of free-living and unrestrained dog populations on human health and well-being in Australia, and understand the framework through which solutions are proposed in the media. Understanding the interpretive frames in media coverage could be helpful for social policy and public health actors to reshape public debates and, ultimately, to promote more effective policies to mitigate the negative impacts of free-living and unrestrained dog populations on health and community well-being [38,39]. 

## 2. Methods 

To identify Australian coverage in text-based news media of issues surrounding the free-living and unrestrained dogs, a search was conducted in Factiva, an international online news database and Proquest Central, a multidisciplinary database with media content from key national Australian and New Zealand newspapers. The following search terms were used: (roaming dog) or (wild dog) or (feral dog) or (stray dog) or (community dog) or (camp dog) for the period 1 January 2000 through 31 December 2019. After several test searches using the Fraser Island dingo colony as a case example confirmed that ‘wild dog’ was the key terminology employed in news reports describing negative impacts of interactions between humans and free-living dogs, particularly health-related issues, the term ‘dingo’ was removed from the search strategy to reduce the number of articles solely focusing on conservation/environmental debates and sporting teams which have a dingo as a mascot. The term ‘dingo’ is used as a synonym for or in direct relation to the term ‘wild dog’ in 83 of the 348 articles in the final sample. A total of 22 of these articles describe attacks by dingoes on K’gari Fraser Island. After limiting content to English language and the region to Australia, more than 3410 items were identified (see Supplementary Figure S1: PRISMA Diagram). In the first screening exercise, 1770 articles were excluded because they were: duplicates (including syndicated republications); incomplete articles; public announcements; or the content did not include dog-related issues. Full-text reports of the remaining 1642 were then downloaded into an Endnote database. We sought to extract articles pertaining to public health issues, including physical attacks on humans, threatening behaviour towards humans, emotional impacts of dog attacks on pets or livestock, and zoonotic diseases. Unique reports of the same incidents and events in different newspapers were included in the study. Because we were focusing on representations of the public health implications and impacts of free-living and unrestrained dogs and associated public policy, a further 1247 articles were excluded during full-text screening because the content was not immediately relevant to the public health dimensions of free-living and unrestrained dog populations described above (for example, reporting only on agricultural or conservation-related wild dog control (1140) or environmental and ecological issues (6) without reference to a health issue or impact). Articles focused on debates about the history and definition of wild dogs (101) were also excluded, after which 348 unique articles remained to be analysed.

The media sample was then read, catalogued manually, and cross-compared by Julie Hall (JH) and Chris Degeling (CD) in order to identify and track prominent concepts, differences, and themes. Next, all authors manually cross-coded a pilot sample (*n* = 20) of the media corpus for specific types of content to confirm and to extend the preliminary thematic analysis. The remaining articles were then coded by JH and Suk Maya Gurung (SMG) for: descriptions of different categories of dogs (including dingoes) and the content and context of their perceived and potential positive and/or negative impacts on people’s health; report of proximal causes and distal drivers for the presence of free-living and unrestrained dogs in and around human settlements/activities and their positive and negative impact on people’s health; descriptions of who or what is responsible for managing the incidence and impacts of free-living and unrestrained dogs; and mention of different interventions and solutions to mitigate or control free-living dog populations. These codes were both emergent and informed by similar media analyses [30,31,40]. 

The results from coding were then tabulated in matrix form and displayed visually as descriptive statistics in charts to aid interpretation. Regular discussions among the authors served to generate additional enquires and to validate insights as they emerged. This approach is consistent with ethnographic content analysis, a qualitative research method for interpreting documents within the context of their use, which enables researchers to generate insights about how documents promote particular ways of understanding, interpreting and responding to an issue or event such as the emergence of a new disease from both numerical and narrative data [41,42]. With these concepts and types of content in mind, our analysis of the 348 articles proceeded through several cycles of immersion and crystallization of insights—a research process comprised of repeated readings and comparisons across and between news-sources, discussions amongst the authors, periods of testing of alternate explanations, and then re-immersion within the research materials [43]. 

Research ethics approval was sought and received from the University of Wollongong Human Research Ethics Committee and New South Wales Aboriginal Health and Medical Research Council. 

## 3. Limitations

This study analyses how journalists describe the public health implications of human interactions with free-living and unrestrained dogs and their populations in Australia. Our analysis only included materials from Australian newspapers and the websites of nation news organisations rather than broadcasts from national and local radio and television news outlets that service the same areas. Incorporating analyses of radio and television broadcasts in the study would be preferable.

## 4. Results 

### 4.1. Sample Characteristics and Settings

The sample was comprised of the text from 348 articles from 94 different newspapers and news websites of large mainstream media organisations—76 were regional newspapers servicing smaller cities, towns and rural/remote areas; 14 were newspapers servicing the capital cities of each of States/Territories; 4 were media sources that were national in scope including *The Australian* Newspaper and the news website for the *Australian Broadcasting Corporation* (ABC). Most of the coverage of the public health impacts of free-living and unrestrained dogs was reported from five media sources; most frequently on *ABC News* websites (38 articles) followed by *The Courier Mail*, Queensland, (22 articles), *Townville Bulletin*, QLD (20 articles), *Northern Territory News* (*n* = 16), and *Centralian Advocate*, Northern Territory, (*n* = 15). 

The majority (56.9%, *n* = 198) of the articles reported on events that occurred in Queensland, 149 of which were published in the 44 newspapers in the sample that service regional Queensland—reflecting the area with the greatest overlap of and contact between wild dog and human populations [4]. 

**Figure 1:**
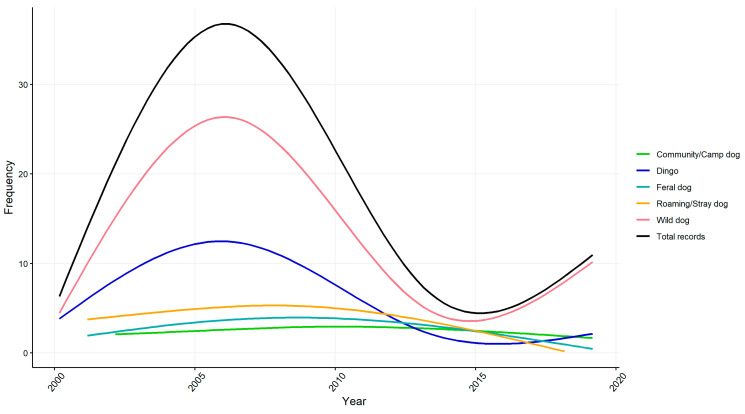
Frequency of articles (by reported dog type) in a study of Australian news sources from 2000 to 2019. Lines are smoothed using a generalised linear model of article counts for each year in order to show the general trend during the study period. Dogs were classified according to terminology used in the news sources.

Figure 1 shows the distribution of articles reporting each dog type throughout the sample period of 2000–2019. Dogs were classified according to terminology used in the news sources. Overall, coverage of issues surrounding the public health impacts of free-living and unrestrained dog populations steadily increases in the first few years of the 2000s before peaking mid-decade, then tailing off and increasing again in 2015. Much of the year-by-year variation in the sample is due to the number of print media reports published on issues relating to wild dogs (a large proportion of which also included use of the term dingo). This two-phase pattern corresponds to the impacts of the Millennium drought on the economies and local ecologies of rural areas in Queensland and a large investment in wild dog control in the early- to mid-2000s, including a raft of studies funded following the founding of the Invasive Animals Cooperative Research Centre in 2005. It is also the case that media interest in different types of stories can wax and wane, as the level of community concern about a specific issue can make it more news-worthy, and, therefore, more likely to be reported in the news as a matter of public interest [36].

### 4.2. Print Media Portrayals of Free-Roaming Dogs as a Social or Public Health Problem 

The key features in the sample of reports on the different public impacts are displayed in Supplementary Table S1 and are elaborated in greater detail below. 

#### 4.2.1. Attacks on Humans by Free-Living and Unrestrained Dogs

Dog attacks on humans are a significant public health problem in Australia—causing injuries, infections and emotional distress. Most people who report bites are bitten by their own dog or an animal that is known to them [6,7]. A description of an attack by an unrestrained dog on a human, a comment drawing attention to the potential for these types of events, or both, was present in nearly one-quarter (80) of the 348 articles in the sample. The category of free-living and unrestrained dogs most frequently associated in print media reports with the incidence and risks of attacks on humans are wild dogs (45 of 80 articles), with roaming/stray dogs and community dogs (also pejoratively described as camp dogs) also sometimes mentioned. These articles are spread evenly across the sample; at least 2 or 3 events are reported in most years (Figure 1). The experiences and perspectives of people who have witnessed or been attacked are a focus (included in 25 of 80 articles). Environmental health and animal control workers are relatively frequently interviewed (20 of 80), with the views of elected officials (ministers, local members, and councillors), and scientists such as ecologists and zoologists occasionally also included.

There are two key types of ‘dog attack’ narrative contained in the media sample, which can be differentiated by where events transpire. Most reported attacks occurred in urban and peri-urban settings; the most common scenario being dog owners attempting to intervene on attacks by a roaming or stray dog on their pet in streets and parks and being bitten or mauled in the process. Unprovoked attacks on humans were a also prominent feature of media coverage of free-living and unrestrained dogs; describing people being bitten when walking the streets or children attempting to pat ‘stray dogs’.

For the second type, a much smaller number of articles describe dog attacks on humans in rural and remote areas. The narrative here differed, with a focus on wild dogs as ‘cross-breeds’ or hybrid animals displaying increasingly brazen and aggressive behaviour towards humans in and around rural properties. Landowners and their families were often depicted as vulnerable to wanton attacks from wild dog packs that have invaded their land and surrounding areas. 

There are patterned differences in how the causes of and solutions to dog attacks are represented and these vary with how the dog is described and where it is encountered. For wild dogs, the increasing size (because of inter-breeding between dingoes and domestic dogs) and aggressive behaviour of individual animals (because of habituation to humans) within these populations is portrayed as a key driver of dog attacks. Most articles that focus on these causes emphasise the effectiveness of controlling free-living and unrestrained dogs through lethal methods (such as culling through baiting and shooting), especially for rural and remote settings. A significant but smaller proportion of articles also point to broader contextual drivers such as increasing contact between human and wild dog populations because of the development and expansion of peri-urban areas. In these articles State/Territory and local governments are portrayed as being responsible for protecting humans and enforcing effective animal control. Notably, despite significant efforts from State and Commonwealth Departments of Agriculture, and peak agricultural industry bodies such as *AgForce*, only a small proportion of articles point to the need for more funding, community empowerment and local co-ordination of efforts to control free-living and unrestrained dog populations. In contrast, when reported events involve a presumably owned but stray or roaming dog on the loose in an urban and peri-urban area, then blame and responsibility tend to be placed on dog owners. Proposed solutions are typically regulatory, punitive, or both, so as to enforce bylaws/regulations relating to dog ownership, and demand that dog owners assume responsibility for controlling their animals. 

#### 4.2.2. The Emotional Impacts on Owners of Wild Dog Attacks on Livestock and Pet Animals 

**Figure 2:**
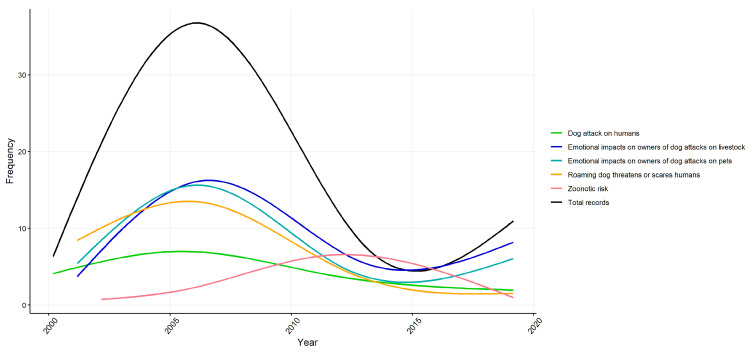
Frequency of articles (by reported impact) in a study of Australian news sources from 2000 to 2019. Lines are smoothed using a generalised linear model of article counts for each year in order to show the general trend during the study period.

Dog attacks on other animals can have human health impacts including the direct emotional trauma of witnessing an attack, and the indirect effects of economic losses and unresolvable concern for the future health and welfare of at-risk livestock and pets on the owner’s well-being [4,7]. More than 200 of the 348 articles in the sample contained mention of the incidence and risks of attacks by unrestrained dogs on other animals such as livestock and pets and the emotional impacts of these events on owners. In the case of attacks on livestock and pets, media attention is increasing again following initial peaks between 2005 and 2010 (Figure 2). In the case of articles describing the human impacts of stock losses associated with wild dog populations, this type of story does not appear in national media but almost exclusively in newspapers serving towns and districts in regional Queensland, Victoria and New South Wales (NSW). Most of these articles focus on the financial losses of the farmers but the brutality of attacks by, often packs, of wild dogs is reported as causing great distress to farmers due to the suffering and mutilation of animals. These impacts are exacerbated by and more visible during times of drought, when stock numbers are lower and the margins of survival, both for stock and the financial health of the farmer, are much tighter. Headlines tend to emphasise the human cost such as ‘Wild dogs leave trail of despair’, and ‘Dog attacks on the rise as weary farmers despair’. When interviewed, farmers describe responses such as ongoing anxiety and sleeplessness due to (often futile) attempts to protect their livestock. The government (i.e., inadequate wild dog management practices) or dog behaviour itself (i.e., relentless, merciless attacks on livestock and pets) are most likely to be ascribed blame and responsibility for the distress experienced by farmers. The need for lethal control measures is almost always emphasised in these articles.

A slightly smaller number of articles report on events and the emotional distress experienced by pet owners (particularly children) whose animals are killed or mauled by ‘roaming’ or ‘stray’ dogs. These events typically occur in urban areas—usually in parks or streets, but also occasionally when a dog has entered a back yard. Interviews with pet owners who recount their attack experience are a common feature of these articles. Because they are portrayed as being responsible for fixing the problem, representatives from local government are usually also interviewed and emphasise the need for responsible dog ownership, with the possibility of dog seizure, fines, and the financial implications of veterinary bills for those who fail to comply. Related to this, because the stray or roaming dog is owned, non-lethal control measures tend to be emphasised. A few articles also focus on the distress experienced by pet owners whose dogs have inadvertently consumed baits intended to control wild dog populations. These unintended or secondary poisoning events are typically framed as a by-product of wild dog management strategies, with blame directed at authorities for irresponsible animal control practices that present risks to uninformed pet owners.

#### 4.2.3. Unrestrained Dogs Threaten or Scare Humans 

More than one-third of the articles (128 of 348) describe events where free-living and unrestrained dogs have threatened or scared humans. Reports of these types of incidents occur in rural, regional and urban settings, from central Launceston in Tasmania to the hinterlands of Townsville in Queensland, peaking just after 2005 before declining then plateauing around 2015. The relative importance given by the media to intimidating encounters is because of the harms caused by dog attacks—but only 11 of these articles include reports of dog(s) physically attacking humans—as described in the previous section. By far the most frequently mentioned type of dog in these articles are ‘wild’ dogs (100 of 128), with some accounts of this type of aggression being displayed by stray/roaming and community dogs. Incidents where people are threatened by an unrestrained dog in public spaces commonly also involve dog attacks on pets (51 of 128) and attacks on livestock (47 of 128). Once again, there is a pattern in how these incidents are reported in the sample. The focus is on individuals describing their experiences of horror and fear. These narratives are often supported with accounts from other local people who have witnessed (often brutal) attacks on pets or livestock which are then discussed in terms of the potential for similar attacks on humans. Articles describe the primary impact of being threatened by an unrestrained dog as emotional trauma which can lead to other consequences such as changes in people’s behaviours and normal routines—most commonly children not allowed to play outside because of concerns about attacks. While descriptions of farmers being threatened by dogs on their properties draw attention to how individual agricultural producers are struggling with the impacts of wild dogs on their livestock, when similar events occur to members of the public in public spaces, follow-up reports of sightings of wild dogs in close proximity to human residential or recreational areas tend to emphasise the threat to humans with headlines such as: ’Wild dogs a danger to pets and children’; ‘Dingo fear grips town as pets mauled’; ‘Dog invasion—Vicious packs roam our suburbs at night’. 

Distinct from reports on other social and public health impacts of unrestrained dogs in public spaces such as bites and zoonotic disease risks, articles describing people being threatened by a dog often explicitly point to the need for governments and agencies to take responsibility for mitigating the threat. Once again, geographic location and the ownership status of the dog determined attributions of blame in these articles. For example, if the unrestrained dog was encountered in an urban or peri-urban area and described as ‘roaming’ or ‘stray’, then the owner was seen as being responsible for any harms. In contrast, when encounters with wild dogs occur in more rural and remote areas, media reports point to a perceived increase in size of populations as a leading cause for encroachment on areas where humans live—the number of wild dogs moving into human settlements being attributed to urban development, drought conditions, or both. Some newspapers in Queensland have even run campaigns to encourage land holders and members of the public to support government efforts at control by making sure they always report the presence of and encounters with wild dogs to authorities. On a much smaller scale, irresponsible owners are also represented as contributing to peri-urban, rural and remote wild dog populations by dumping domestic dogs, with hunters and transient fly-in-fly-out workers specifically mentioned. Solutions proposed were equally oriented to lethal (baiting and culling) and non-lethal forms of population control (trapping and neutering) with wild dogs the preferred target for the former, and stray and otherwise owned but at-large dogs the latter.

#### 4.2.4. Zoonotic Risks to Humans from Free-Living and Unrestrained Dogs

Dogs can carry infectious pathogens that can also infect humans to cause disease. Globally, zoonotic infections such as rabies virus (which is exotic to Australia) cause significant human mortality and burdens of disease [5,7]. Less than one-tenth of the articles in the sample point to the incidence and risks of zoonotic diseases as a public health impact of free-living and unrestrained dogs. Reports that mention zoonotic risks occur most frequently in Queensland local or community newspapers with media interest concentrated between 2005 and 2009. Zoonotic risks are mainly discussed as a ‘wild dog’ issue. There are a couple of mentions of ‘non-specific’ infectious disease risk from community dogs—this coverage being localised to the Northern Territory. Most of the articles are about echinococcosis due to the tapeworm parasite *Echincoccus granulosis*, which is represented as serious and sometimes fatal infection passed on to people through contact with dogs, as well as being an infectious risk to pets and livestock. Because the non-sylvatic lifecycle of *E. granulosis* can involve sheep and cattle, the views of infectious disease researchers, zoologists and representatives of agricultural industry groups are frequently included. The bulk of reports that include discussion of risk of echinococcosis from ‘wild’ dogs appear in 2008–2009, with a later spike in interest during the period 2018–2019. Urban sprawl and increase in the size of the wild dog population are described as the key causes. Because the dogs are potential hosts of *E. granulosis*, the increasingly contact between wild dog populations and humans in peri-urban areas is clearly identified as an emerging public health risk. Culling of wild dogs is again presented as an element of the solution to this issue, but people are also told to take responsibility for protecting themselves through adopting hygiene behaviours, i.e., awareness, hand washing, and not encouraging wild dogs by leaving rubbish out. In contrast to the other public health impacts of free-living and unrestrained dogs described in the media corpus, there is a much greater focus on community empowerment around this issue, which is closely aligned with the need for greater public awareness of the risks.

#### 4.2.5. The Public Health Impacts of Community Dogs on Aboriginal and Torres Strait Island Peoples 

The shared environment of Aboriginal and Torres Strait Islander people and co-located dog populations has caused concern among Australian public health authorities for decades, with zoonotic disease and bite prevention a particular focus [5,10]. Only a small subset of the articles in the sample (23 of 348) focused on the public health risks and impacts of community dogs in First Nations communities in the Northern Territory—particularly around Alice Springs and remote outstations. While the impacts of wild and roaming/stray dogs dominate coverage in regional media, concerns about the public health implications of community dogs are prominent in national media sources—most conspicuously the Australian Broadcasting Corporation. Although the experiences of people affected by the impacts of dog attacks and aggressive behaviour are a prominent feature of media coverage of these issues, only 2 articles of the 23 in the sample that describe community dogs as a public health problem include interviews with dog-owning community members—both of which also describe a positive role for dogs in the day to day activities of the community. The remainder of the articles tend to focus on the perspectives of local authorities who emphasise the negative impact of community dogs—especially canine humbugging (begging for food), zoonotic risks, the lack of community hygiene and the relative health and poor welfare of animals. The types of people who get to speak to these specific issues in the media are usually service providers in local councils. The focus of these interviews is on the number of unrestrained ‘camp’ dogs living in the community, with some elaboration about lack of funding for and community uptake of control efforts—either veterinary services or through bylaws. Only a handful of articles ascribe responsibility for the problems caused by community dogs to dog owners. A key concern framing these discussions is that there is a lack of community trust in dog control measures. Yet the most prominent solutions proposed in the media are veterinary interventions and regulatory in nature, the effectiveness of which both require broad community support for the proposed actions. 

## 5. Discussion 

The public health impacts of free-living and unrestrained dogs in Australia have complex causes that intersect at the nexus between human and canine behaviour, agricultural and land management practices, local bylaws, and efforts to conserve ecological systems. However, media coverage of public health issues pertaining to free-living and unrestrained dogs is usually locally focused, and, particularly in the case of wild dog populations, driven by first-hand accounts of problematic human-animal encounters, which at best fragments, but more frequently, erases this complexity. Urban sprawl, the increasing use of natural areas as sites of human recreation, and the effects of droughts on the range and size of wild dog populations, mean that the situation is evolving. As human and wild dog populations increasingly overlap, there is a greater risk of public health impacts in new sites and locations [44]. Yet media discussion of this issue is dominated by the need to “control” dogs—within which reports of attacks on pets and livestock are used to amplify the threat that free-living, and, therefore, uncontrolled dogs can pose to humans. Consistent with the longstanding cultural tropes and logics that surround rabid animals [45], in Australian printed news media discourse, free-living and unrestrained dogs are regarded as an existential threat to individual safety and community well-being. The level of threat is amplified when the interface between free-living dogs and humans involves greater competition for resources—particularly during periods of drought.

Within this worldview, the livelihood and well-being of farmers are under a sustained assault from elusive but destructive packs of wild animals and the safety of ordinary members of the public and their pets are threatened by ever more dangerous types of dogs, not only in ‘wild areas’ such as National Parks, but also on the streets and in other public spaces in and around their communities. The level of risk to individual safety and community well-being is coded into media reporting in the way the offending dog(s) are described, the location of encounters, and implied ownership status. Owned stray and roaming urban dogs appear at one end of the spectrum of threats to public order and the uncontrolled, unowned, and, thereby, more difficult to manage categories of peri-urban feral and wild dogs at the other. With regard to this, the terms wild dog and dingo are interchangeable and discursively loaded such that Australian public attitudes toward “dingoes” are largely positive but attitudes toward “wild dogs” are entirely negative [24]. Also notable, scientific studies of dog ecology and control funded by livestock production organisations are more likely to employ ‘wild dog’ terminology [25]. The effect is that in discourse and practice, wild dogs are killable, whereas dingoes should be protected [32]. Unsurprisingly, programmatic government actions such as culling (for which the term trapping is a euphemism) is almost exclusively positioned in the media sample as the clear solution to the impacts of wild dog populations in rural settings. Even though evidence of the effectiveness of lethal control at reducing impacts of wild dogs on human interests is limited, and sometimes conflicting [46,47].

Our findings are consistent with previous research in Calgary, Canada that shows that journalists portrayed dog bites as social deviance and social in nature, so as a policing rather than public health problem, while framing out the role of social inequalities in dog bite risks [30]. Following on from this, understanding the incidence of free-living and unrestrained dogs as a law and order problem limits opportunities to evaluate the true extent of their health-related impacts. Social and law and order problems, such as substance abuse or family violence, for example, are only integrated into broader health policies and documented and reported as the outcomes of such when there is widespread recognition of their significance to public health [48,49]. Analyses of public hospital reports to the Australian Institute of Health and Welfare indicate that the highest rates of dog bites are recorded for the Northern Territory, followed by Tasmania, the second most underserved State in the commonwealth [6]. This is consistent with other high-income countries such as Canada and the UK, where most problematic human–dog encounters occur in inadequately served areas, both in terms of access to health care and local government services [50,51]. If the patterns of injury are the same as the UK and North America, then the majority of dog bites in Australia are from pet dogs and treated in primary care [51,52]. However, data on the prevalence of dog bites in Australia—let alone the type of dog involved—is not systematically collected or consistently reported. The result is that relevant policy actors, the media, and by extension, the broader public, are blind to the magnitude of issues surrounding dog aggression, and the extent to which the incidence of this and other dog-related public health impacts overlap with social disadvantage. Therefore, the direct impacts and consequences of dog attacks and other forms of aggression are not high on the agenda for public health authorities, who consider animal control to be in the grey area between community policing and environmental health issues.

Our analysis suggests that when thinking about the impacts of free-living and unrestrained dogs, it is also important to consider the potential implications of dog aggression on physical and psychological health. Acknowledging that news media tend to highlight the personal impacts of traumatic events, people can feel unsafe and stressed when in public spaces because they have previously been attacked or threatened by a dog, or have witnessed a dog attack on pets or livestock [53]. Looking at wild dog attacks on livestock, in particular, the impacts on farmers are typically measured and reported in the media and scholarly literature in economic terms [54], but recent studies have documented the mental health impacts of wild dog attacks, suggesting that “critical incidents” can lead to both individual and community trauma [55]. In addition to the mental health impacts, there are financial impacts caused by wild dog attacks on livestock. These impacts, and the reporting of them, appear to be heightened during times of drought conditions. While financial stress can have health consequences, further evidence of the health impacts of emotional distress caused by stock losses through uncontrolled wild dog populations is lacking, which means that news media, policy actors and the broader public lacks a frame to understand and respond appropriately. Noting that 1140 excluded items of the original 3412 articles identified in our search related directly to wild dog control, this aspect of public discussion is likely greater than is represented here.

Public and institutional visibility is key to prompting an organised response to a problem [56]. Who does and does not get to speak about an issue in the media shapes public perceptions, which influences the broader acceptability and scope of what are seen as being appropriate responses and solutions [57]. This effect can clearly be seen in discussions of Community dogs in the media sample. Indigenous and non-Indigenous members of communities in Australia have different perceptions and practices surrounding dog ownership. However, media and public health discourses emphasise the need for cosmopolitan norms of responsible dog ownership, while ignoring the positive roles free-roaming community dogs can play in promoting social cohesion and human health and well-being [10,58]. Dog-owning members of Indigenous communities rarely speak about these issues in the media. Conversely, when the attack is by a wild dog on owned livestock or pets, the accounts of bystanders, witnesses and the owners of the injured or dead animal are emphasised to draw attention to what happens when control and order break-down. This near inversion of the platform given to owners of controlled pets and unrestrained community or camp dogs is conceivably an extension of the settler colonial logics that continue to shape institutional approaches to the norms of dog keeping in many Aboriginal and Torres Strait Islander communities [59]; and, more broadly, how some ideas are given prominence over others in public discourse so as to sustain cultural and political consent to eradicate animals that are problematic or otherwise unwanted by those with the power to pursue these ends [32].

Turning once again to policy solutions, peri-urban and rural populations are becoming increasingly diverse. There is a plurality of values of importance to the place of free-living dogs in Australia, including concerns for animal rights and welfare, social equity, and environmental sustainability. Under these conditions, the prioritisation of public discourses that focus on controlling dog populations limit the potential for the adoption of more integrated and sustainable measures. In the absence of One Health thinking, local solutions to interrelated problems such as increasing contact between human and wild dog populations and dog aggression may be developed separately or in an ad hoc manner. Approaches to dog population management and control in Australia are well informed by scientific forms of expertise such as species ecology. Yet within this context, the focus of much of the media coverage on wild dog issues continues to be the vulnerability and powerlessness of individuals to effect change, which necessitates greater actions by government to restore and maintain order to protect individuals and their communities. As more Australians are embracing living and spending time in places which are more closely entwined with wilder landscapes, there is a clear expectation in media discussions that local authorities need to keep them safe. When ecologists, zoologists, rangers and animal control officers have an opportunity in the media to share their perspectives on the risks of wild human–dog interactions in these settings, the solution is not regulatory, but a need for individuals and communities to adopt appropriate behaviours and activities. 

## 6. Conclusions 

Media reporting of the negative impacts of free-living and domestic dogs is patterned according to context and location, promoting and reinforcing a public discourse in which both the causes and actions to prevent such impacts are similarly patterned. This simplification and fragmentation of discourse limits recognition of the underlying complexity of intertwined human and canine ecosystems, resulting in calls for simplistic solutions that ignore the potential benefits of a One Health approach. Rather than requiring government actions, mitigating the evolving public health risks and impacts of free-roaming dogs requires ground-level health promotion activities such as public education and awareness raising to prompt individuals to act responsibly. Australian local governments are well positioned to work with local health authorities to play a more prominent role in community adaptation to changes in environmental health risks [60]; the benefits of community-based approaches have been demonstrated in other contexts [61,62]. Yet without developing evidence and appropriate advocacy, the non-zoonotic impacts and issues surrounding free-living and unrestrained dogs could continue to remain solely a matter of public order in public and policy discourses, such as to perpetuate the failure to systematically address the broader ripple effects of human–wild dog conflicts and accommodations and their public health implications.

## Figures and Tables

**Table 1:** Definitions of dog types in the context of a media analysis of the representations of dogs as an emerging public health issue in Australian newspapers.

Dog Type	Description
Community/Camp dog	A domestic dog living in a remote Indigenous or Torres Strait Island community. The dog can be owned or unowned, and free-roaming or confined to a house and yard.
Dingo	Wild dog that is native to Australia.
Feral dog	A domestic dog that is unowned and free-living (without human support). The term implies that the dog is living in rural or remote locations, outside human settlements
Roaming/Stray dog	A domestic dog that is currently or previously owned (and unwanted) and is free-roaming. The term implies that the dog is living in human settlements
Wild dog	A term used in Australia to describe different types of free-living dogs and unowned dogs, including dingoes, feral domestic dogs and dog–dingo hybrids.

## Data Availability

Data is available on request.

## Supplementary Materials

The following are available online at https://www.mdpi.com/article/10.3390/ijerph18115807/s1, Figure S1: Search terms and PRISMA diagram, Table S1: Key features of reports of the public health impacts of free-living dogs in Australian newspapers.
