# Supplementary material for: Representations of Free-Living and Unrestrained Dogs as an Emerging Public Health Issue in Australian Newspapers
Source: Int J Environ Res Public Health. 2021 May 28;18(11):5807. doi: 10.3390/ijerph18115807 (PMC8198982; doi:10.3390/ijerph18115807)
Supplement: Supplementary file 1 [file ijerph-18-05807-s001.zip › ijerph-1183034-supplementary.pdf]

**Figure S1. Search terms and PRISMA diagram**

"roaming dog" OR "stray dog" OR "feral dog " OR "wild dog " OR "camp dog" OR "community dog"

01/01/1990 to 31/12/2019. English Language, Region=Australia, Newspapers

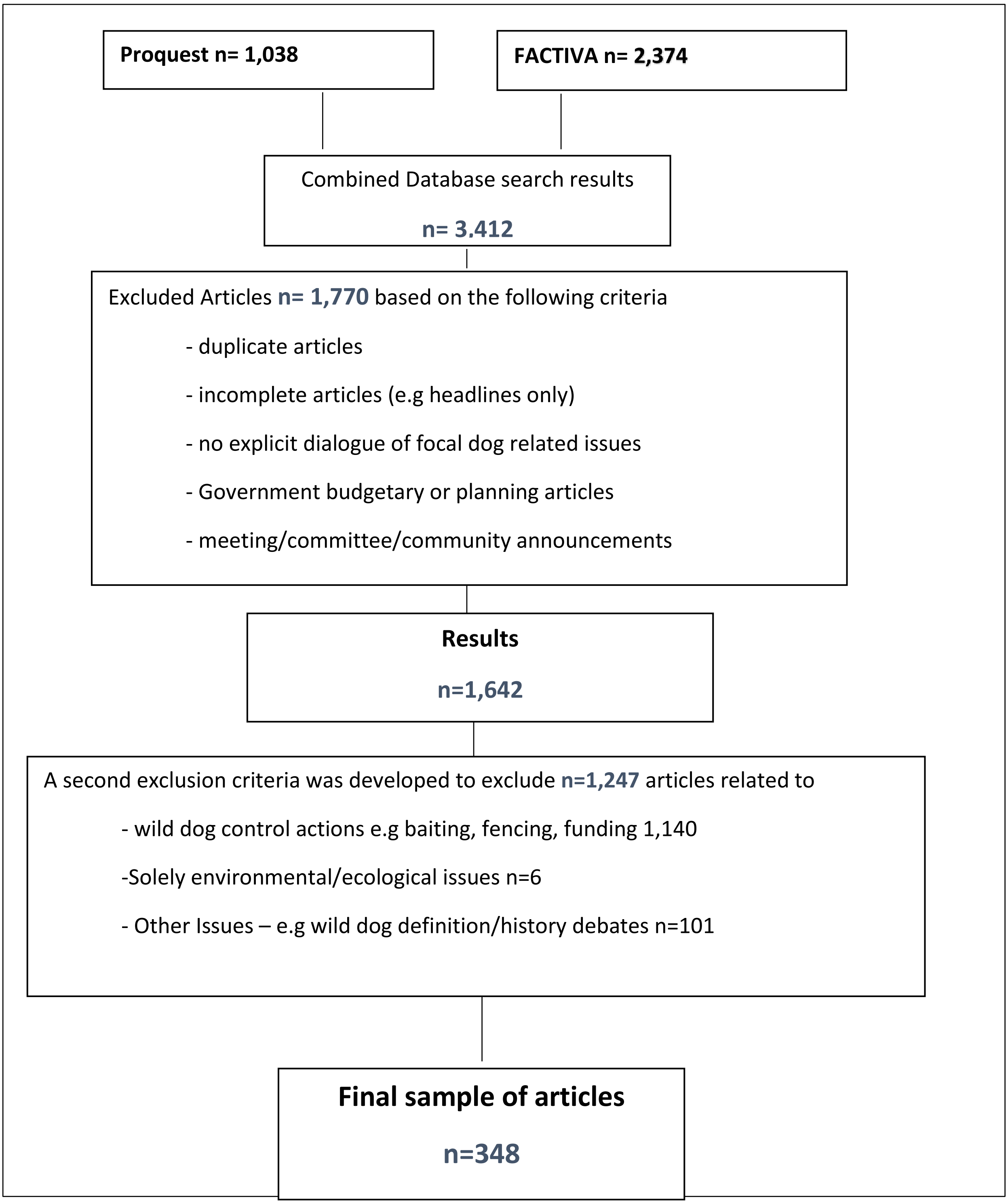

**Table S1**: Key features of reports of the public health impacts of free-living dogs in Australian newspapers

| Reported Public Health Impact | Number of reports | Most frequent media source | Category of dog most frequently implicated* | Most frequently reported setting |
|---|---|---|---|---|
| **Attacks on humans by free-living dogs** | 80 of 348 articles | • 38 reports in local/regional newspapers<br>• 30 reports in provincial newspapers<br>• 12 reports in national media | • Wild dogs (45)<br>• Roaming/Stray dogs (14)<br>• Community/Camp dogs (6)<br>• Feral dogs (5) | Urban and peri-urban areas |
| **Emotional impacts of wild dog attacks on livestock and pet animals** | 203 of 348 articles | • 123 reports in local/regional newspapers<br>• 8 reports in provincial newspapers<br>• 72 reports in national media | • Wild dogs (179)<br>• Roaming/Stray dogs (28)<br>• Feral dog (15)<br>• Community/Camp dogs (3) | • Attacks on livestock in rural and remote areas.<br>• Attacks on pets in urban areas |
| **Unrestrained dogs threaten or scare humans** | 128 of 348 articles | • 77 reports in local/regional newspapers<br>• 35 reports in provincial newspapers<br>• 14 reports in national media | • Wild dogs (100)<br>• Roaming/Stray dogs (17)<br>• Feral dogs (14)<br>• Community/Camp dogs (5) | Occurs in all settings |
| **Zoonotic risks to humans from free-living dogs** | 21 of 348 articles | • 17 reports in local/regional newspapers<br>• 2 reports in provincial newspapers<br>• 2 reports in national media | • Wild dogs (18)<br>• Community/Camp dogs (5) | Mainly peri-urban areas; some in First Nations' communities |
| **Public health impacts on Aboriginal and Torres Strait Island peoples** | 23 of 348 articles | • 12 reports in local/regional newspapers<br>• 5 reports in provincial newspapers<br>• 7 reports in national media | • Community/camp dogs (23) | First Nations' communities |

* An article can mention more than one type of dog
